# Vitamin d deficiency with high parathyroid hormone levels is related to late onset SEPSIS among preterm infants

**DOI:** 10.1186/s12884-022-05334-2

**Published:** 2023-01-13

**Authors:** I. Tofe-Valera, J. L. Pérez-Navero, J. Caballero-Villarraso, M. D. Cañete, R. Villa-Jiménez, M. J. De la Torre-Aguilar

**Affiliations:** 1 Neonatology, Department of Pediatrics Unit, Reina Sofia University Hospital. Córdoba. Maimónides Biomedical Research Institute of Córdoba (IMIBIC), Córdoba, Spain; 2 Department of Pediatrics, Unit of Pediatric Research, Reina Sofia University Hospital, Maimonides Institute of Biomedical Research of Cordoba (IMIBIC), University of Córdoba, Córdoba, Spain; 3 CIBERObn Center for Biomedical Research on Rare Diseases (CIBERER), ISCIII, Madrid, Spain; 4 School of Medicine, University of Córdoba, Córdoba, Spain; 5 Pediatric Research Unit, Maimónides Institute for Biomedical Research of Córdoba (IMIBIC), Ave. Menéndez Pidal 7. P. C. 14004, Córdoba, Spain; 6 Department of Biochemistry and Molecular Biology, Clinical Analyses Service, Reina Sofia University Hospital, Córdoba, Spain; 7 Associate Professor at Córdoba University (UCO), Córdoba, Spain

**Keywords:** 25(OH) D levels, Vitamin D deficiency, Preterm infants, Low birth weight, sepsis, Parathyroid hormone

## Abstract

**Summary:**

Preterm infants (PTs) are at greater risk for vitamin D deficiency, which relates to the possibility of a higher incidence of comorbidities. Our goal was twofold, 1) to monitor vitamin D, calcium, phosphorus, parathyroid hormone (PTH), matrix metalloproteinase-8 (MMP-8) serum levels at three-time points during hospitalization, and 2) to assess the association between 25-hydroxyvitamin D (25OHD) levels and outcomes in PTs.

**Methods:**

We carried out a follow-up on 50 Caucasian PTs ≤ 32 weeks of gestational age (GA) and/or ≤ 1500 g birth weight at 28 days and at 4 months. PTs were divided into two subgroups for tests of association with clinical outcomes based on vitamin D deficient infants 25(OH) D cord blood levels: ≤ 20 ng/ml). At an initial stage, 25(OH) D levels were determined in maternal/preterm blood samples and were compared to full term delivery infants.

**Results:**

There were no differences in 25(OH) D serum levels at birth when comparing PTs to term infants, or regarding maternal levels. A strong positive correlation was detected between maternal and neonatal 25(OH) D serum levels among PTs and term infants (r: 0.466; *p* < 0.001). Neonates with vitamin D deficiency did not present a higher incidence of comorbidities. PTs were classified in two subgroups based on vitamin D and PTH (group 1: vitamin D < 20 ng/mL and PTH > 60 pg/mL; group 2: vitamin D > 20 and PTH < 60 pg/mL). The PTs in group 1 showed a higher incidence of LOS (RR: 2; 95% CI: 1.31–3.55). No relationship was observed between MMP-8 serum levels and the incidence of sepsis.

**Conclusions:**

This study did not find any evidence of an increase in preterm birth risk related to vitamin D level at birth. Vitamin D deficiency by itself is not associated with a higher incidence of comorbidities. However, the binomial vitamin D-PTH must be taken into consideration.

## Introduction

Preterm infants (PTs) ≤ 32 weeks of gestational age (GA) and/or ≤ 1500 g birth weight have a higher risk for vitamin D deficiency as a result of a high prevalence of vitamin D deficiency in the mother during pregnancy, insufficient sun exposure during hospitalization and difficulty in ensuring adequate enteral nutrition [1]. Serum levels of 25(OH) D are the most widely known marker of vitamin D status, whose function is to regulate calcium and phosphorus homeostasis.

There is increasing evidence regarding the non-classical function of vitamin D and its pleiotropic effects, not only on bone metabolism but also on the proper functioning of the organ systems. Vitamin D may become, thus, a vital modifying factor for certain diseases, such as cardiovascular diseases [2], insulin resistance, metabolic syndrome [3], allergy [4], autoimmune disorders [5] and different types of cancers [6]. Similarly, vitamin D is pivotal for the innate immune system, promoting the production of defenses which have antimicrobial and antiendotoxin activities [7–10]. Moreover, low maternal vitamin D status is associated with a higher risk for preeclampsia, gestational diabetes mellitus and other gestational diseases. Likewise, several negative consequences for the fetus have been reported, including fetal growth restriction, increased risk of preterm birth and a changed susceptibility for later-life diseases [11].

Recent reports have highlighted the role of vitamin D in promoting the normal function of the innate and adaptive immune systems [12]. However, despite late advances in risk assessment and sepsis prevention, vitamin D deficiency remains a global health problem. In fact, there are numerous risk factors for sepsis and mortality risk which appear to decrease as 25(OH) D serum levels increase [13, 14]. Studies in hospitalized adult patients have suggested a relationship between vitamin D status and later prognosis and have indicated that 25(OH) D serum levels < 20 ng/mL are linked to adverse outcomes [15].

While vitamin D deficiency is considered a very common condition worldwide, currently there is no consensus on the optimal levels of 25(OH) D [16, 17]. The Endocrine Society defines vitamin D deficiency as 25(OH) D serum levels < 20 ng/mL and suggests that the target treatment should be to reach > 30 ng/mL [18]. On the other hand, the American Academy of Pediatrics (AAP) considers deficiency when values are between 5 ng/mL and 10 ng/mL [19]. Furthermore, the existing definition for vitamin D adequacy in PTs for health outcomes remains controversial, and an optimum vitamin D status is based on targeting 25(OH) D serum levels > 30 ng/mL in adults. Vitamin D levels related to the normal inflection point of PTH should be interpreted as an optimal situation and a marker of sufficient value of 25(OH) D serum levels [20, 21].

The expression and activity of matrix metalloproteinases (MMPs) is regulated by vitamin D. MMPs are a family of nine highly homologous Zn (++)-endopeptidases that cleave collectively most of the constituents of the extracellular matrix. MMPs function as important modulators of immune responses. However, an uncontrolled MMPs activity may derive into tissue destruction [22]. MMPs have been suggested to be involved in the pathogenesis of sepsis and septic shock [23]. The expression and activity of MMPs is normally low but increases in many pathophysiological conditions, such as infection. In addition, it has been reported that 25(OH) D in cord blood correlates with inflammatory markers MMP-8 and C-reactive protein (CRP). These findings may prove the diverse immunomodulatory functions of vitamin D in the innate immune response of neonates [24]. Despite advancements in neonatal care, sepsis remains a significant cause of morbidity and mortality globally. Therefore, primary preventive strategies, an early diagnosis and receiving optimal antimicrobial therapy are crucial to prevent death or disability in PTs.

The current study hypothesized and aimed to evaluate if low 25(OH) D serum levels are related to prematurity and to a higher risk for developing comorbidities. Accordingly, we have undertaken a three-time points study to find evidence on possible associations between the markers studied and the pathology presented by PTs at birth, 28 days of life and at 4 months.

## Materials and methods

### Study design. Case-control

We conducted a case-control study, at an initial stage, with a cohort of 50 term infants and their mothers (Control Group) and 50 PTs ≤ 32 weeks’ GA and/or ≤ 1500 g birth weight and their mothers (Case Group) and measured 25(OH) D serum levels at birth.

**Fig. 1:**
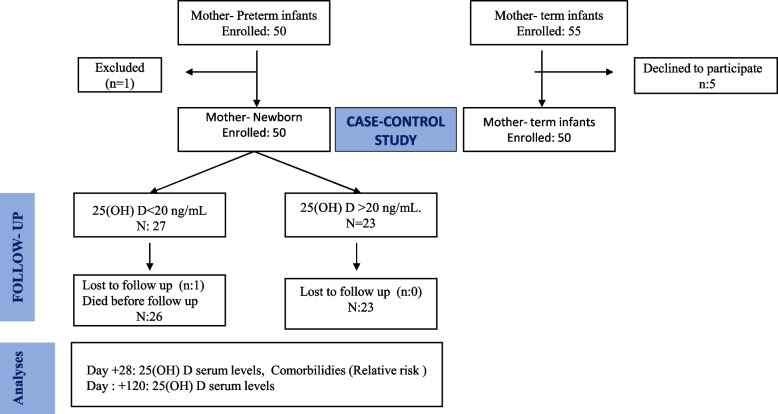
Flow Diagram

All the neonates in both groups were selected consecutively. Figure 1 shows the flow diagram of participants in the study. The inclusion criteria for this study were: female and male term infants and PTs that had ≤32 weeks’ GA and/or ≤ 1500 g birth weight. Participants were excluded if they met the following exclusion criteria: they did not fulfil the age and weight at birth established, they or their mothers were not Caucasian, and/or if PTs presented 1) mortality before 36 weeks, 2) Chromosome abnormalities, 3) Genetic anomalies or 4) Congenital malformations. The control group encompassed healthy term infants with adequate birth weight.

### Longitudinal study design

We carried out a follow-up on PTs at 28 days and at 4 months and divided them into two subgroups for tests of association with clinical outcomes based on 25(OH) D cord blood levels: ≤ 20 ng/ml and > 20 ng/ml, following the criteria provided by the Endocrine Society Clinical Practice Guidelines [18].

Consecutive sampling was performed at their hospitalization in a neonatal intensive care unit (NICU) in conjunction with the Clinical Analysis Service (Reina Sofia University Hospital, Córdoba, Spain). Our study was carried out in accordance with the Declaration of Helsinki and approved by the Institutional Hospital Ethical Committee. The participants involved were incorporated after all the inclusion criteria were fulfilled, and informed written consent was obtained from their parents or legal guardians. Confidentiality of all personal information was protected, and access to medical data was provided conforming to the hospital ethical standards.

Demographic and clinical data, including data on pregnancy (vitamin D supplementation) and delivery (season of birth), were collected from the mothers, term infants and PTs. Among PTs, Clinical Risk Index for babies score (CRIB I and II), a morbimortality score as a predictor of hospital death and intraventricular hemorrhage in very low birth weight PTs, was obtained. Pathologies related to prematurity were noted: sepsis, hyaline membrane disease, bronchopulmonary dysplasia (BPD), intraventricular hemorrhage, periventricular leukomalacia, retinopathy, and necrotizing enterocolitis (NEC). BPD diagnosis and severity was based on the need for oxygen for 28 days and 36 weeks’ postmenstrual age [25]. Late onset sepsis (LOS) is defined as the development of signs and symptoms of severe sepsis after 72 hours of life (blood culture, complete blood cell count, C-reactive protein and procalcitonin) [26].

### Vitamin D supplementation

Early vitamin D supplementation in PTs was started at 1000 UI/day and administered, if feasible, by an orogastric tube prior to feeding at 48 hours. Parenteral nutrition was started within the first hours of life at a daily dose of 120 IU vitamin D. Parenteral nutrition in the study center is personalized since the first 24 hours of life. Rates of vitamin D daily administered intravenously via parenteral nutrition were 120 IU. In order to calculate the total amount of vitamin D, the type and quantity of lactation were considered. At discharge, PTs received an intake up to 400 IU, and 25(OH) D serum levels were monitored at 4 months.

### Blood sampling and biochemical analysis

Blood samples from neonates were obtained from the cord blood and drawn into BD Vacutainer® SST (BD) tubes. At 28 days of life and at 4 months, the blood samples of the PTs were obtained from a peripheral vein as part of the routine protocol followed in the unit to monitor metabolic status. No extra blood volume was required. Maternal blood samples were obtained from a peripheral vein in the delivery room after the mothers had given informed written consent. The blood collected into these tubes was allowed to clot in the cold for 30 min prior to centrifugation at 1500 x g for 10 min at 4 °C. Once centrifugated, the samples were separated into aliquots and frozen at − 80 °C until analysis. Serum levels of 25(OH) D were measured by Radioimmunoassay (RIA), using an immunological test kit (LIAISON® 25 OH Vitamin D TOTAL Assay; DiaSorin), following the manufacturers’ instructions with the PACKARD Copper II E5005 gamma counter analyzer.

Parathyroid hormone (PTH) was measured by immunoradiometric assay by our CAS. MMP-8 were quantified by a semi-automated TRITURUS®-200 open-configuration kit that uses Xmap technology as a basis, designed for determinations by the sandwich ELISA (enzyme-linked immunosorbent assay) technique, using a 450-nm microplate reader with capacity for automatic dispensing of samples/reagents and reading of results directed by computer.

### Statistical analysis

The sample size was calculated using the GRANMO (2012) software, accepting an alpha risk of 0.05 and a beta risk of 0.2 in a two-sided test. Twenty-four subjects in the deficient 25(OH) D subgroup and 23 in the non-deficient subgroup are necessary to recognize a relative risk (RR) greater than or equal to 2 as statistically significant. A proportion in the non-deficient group was estimated to be 0.45. A drop-out rate of 0% was anticipated.

The data were expressed as mean ± SE. For the data that fit a normal distribution the Shapiro-Wilk test was utilized. Homogeneity of variance was estimated using the Levene’s test. Mean values for continuous variables with normal distribution were compared by the Student’s t-test for unpaired samples, and by the Mann-Whitney U test for data with asymmetric distribution. Categorical data were analyzed by χ2 or the Fisher’s exact test. Relative risk and confidence interval (CI) 95% were considered for appearance of comorbidities based on vitamin D levels at birth. Associations between potential determinants and 25(OH) D levels at birth were investigated by using a multivariate linear regression model.

Vitamin D levels were compared at times 0 and at 28 days with a breakdown by comorbidities using repeated-measures ANOVA with Sidak correction for post-hoc analysis and were adjusted for GA and gender as covariates. The logistic regression analysis, adjusted for potential confounders, was performed to determine the effect of blood 25(OH) D concentrations at 28 days on the risk of LOS.

Correlations between the different variables were performed using the Pearson’s rho test. All tests were two-tailed, and a *P*-value < 0.05 was considered statistically significant. Statistical assessment was carried out using the SPSS v.27 (Ecosoft, Indianapolis, IN, USA).

## Results

Fifty consecutive PTs ≤ 32 weeks’ GA and/or ≤ 1500 g birth weight and 50 term infants with their respective mothers were enrolled in the present study. Most PTs’ mothers (48/50) were administered corticosteroids (complete course, Betametasone im 12 mg/24 h/2 days. None of the mothers and neither PTs nor term infants were supplemented with vitamin D during pregnancy. All the PTs started on early vitamin D supplementation with amounts as specified in the Materials and Methods section.

### Study design. Case-control

Demographic characteristics are displayed in table 1. No differences were observed between the control group and the PTs group in relation to the age of the mothers, gender of participants and season of birth.

**Table 1:** Demographic characteristics of the study population

		Term Infants N:50	Preterm Infants N:50
**Maternal age (years)**		32.6 ± 5.9	33.1 ± 5.3
**Gestational age (weeks)**		38.7 ± 1.14	29.9 ± 2.4^a^
**Newborn weight (gr)**		3294 ± 528	1311 ± 307^a^
**Length (cm)**		49.7 ± 3	38.8 ± 3.5 ^a^
**Cefalic perimeter (cm)**		34.9 ± 2.5	27.4 ± 2.3^a^
**Gender (male)**		20(40%)	24 (49%)
**Season**	Spring	20(40%)	11(22%)
	Summer	6(12%)	15(31%)
	Autumn	7(14%)	8(16%)
	Winter	17(34%)	15(31%)

Data are given as mean ± standard deviation and absolutes frequencies (percentages). *P* values were obtained from the Mann-Whitney U-test, Student’s T test, or the Fisher exact test, as appropriate. ^a^: *P* < 0.05 value between prematurity and term infants

Biochemical data are shown in table 2. There were no differences in 25(OH) D serum levels at birth when comparing PTs to term infants, or regarding maternal levels.

**Table 2:** Maternal and neonatal 25-hydroxyvitamin D (25-OH) D levels and biochemical markers in prematurity and term infants

	Term Infants N:50	Preterm Infants N: 50
**25(OH) D Mothers (ng/mL)**	9.78 (24.20)	10.49 (22.15)
**25(OH) D Neonates (ng/mL)**	13.2 (7,85)	18.6 (13.62)
**Calcium (mg/dl)**	11(6)	10.15(7.4)^a^
**Phosphorus (ng/ml)**	6.2 (8.1)	5.5 (9.3)^a^
**Osteocalcin (ng/mL)**	31.9 (20.27)	61(104.6)^a^
**PTH (pg/mL)**	2.25 (3.55)	49.1(84.68)^a^
**MMP8 (ng/mL)**	2692(2104.8)	500 (70.3)^a^

PTH: Parathyroid hormone. MMP-8: Matrix metalloproteinase-8. Data are expressed as medians (interquartile ranges). Statistical significance obtained by Mann–Whitney U test. ^a^: P < 0.05 value between prematurity and term infants

**Fig. 2:**
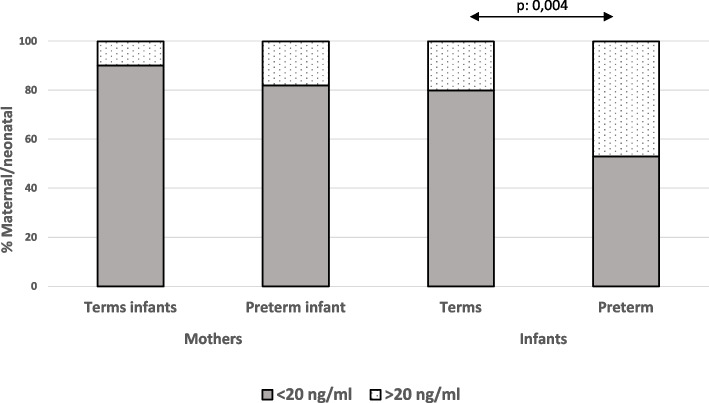
Maternal and neonatal percentage in both groups, subdivided according to a cut-off point of 20 ng/ml for 25(OH) D levels at birth

Mothers and neonates in both groups were subdivided according to a cut-off point of 20 ng/ml for 25(OH) D. We observed that the percentage of PTs with 25(OH) D > 20 ng/mL was significantly higher compared to the group of term neonates (p: 0.006) (Fig. 2).

There was a positive correlation concerning 25(OH) D serum levels of mother/ infant (r: 0.466; *p* < 0.001) in both groups. A regression lineal model was conducted to identify factors associated with vitamin D levels at birth and we found that 25(OH) D levels were dependent on the mother’s levels (Table 3).

**Table 3:** Multivariate linear regression on Neonatal 25(OH) D levels at birth

	ß Coefficient Unadjusted	Multivariable Model	P value	95%CI
**Constant**	10.147		0.001	4.128–16.167
**Maternal** **25(OH) D levels**	0.822	0.471	0.001	0.336–1.309

Multivariate linear regression models with B coefficient, 95% confidence interval (CI). Multivariate Model: the model was adjusted for gestational age, newborn weight, gender and season

Regarding the biochemical data analyzed, higher levels of osteocalcin and PTH were observed in the PTs group. MMP-8 levels were significantly higher in the term infants group. Despite we found significant differences concerning the levels of calcium and phosphorus among the two groups, such did not have clinical relevance as values were still within the normal range (Table 2).

No correlation was observed between levels of osteocalcin (r: 0.180; p: 0.097), PTH (r: 0.065; p: 0.545), MMP-8 (r: 0.05; p: 0.625) calcium (r: 0.024; p: 0.816), or phosphorus (r: − 0.039; p: 0.721) with vitamin D. On the other hand, the biochemical data were related to GA (osteocalcin r: − 0.446; *p* < 0.001; PTH: r: − 0.472, p < 0.001; MMP-8: r: 0.386; p < 0.001), calcium: r: 0.328; p: 0.001) or phosphorus (r: 281; p: 0.009) levels at birth.

### Longitudinal study design

The longitudinal follow-up on the PTs group (Table 4) showed that 25(OH) D serum levels remained constant until day 28 to later have a significant increase at 4 months. Table 4 also describes the course of PTH levels, osteocalcin, and MMP-8 over time.

**Table 4:** 25(OH) D, PTH, Osteocalcin and MMP-8 serum levels in Preterm Infants: change from baseline to 28 day and 4 month

	Baseline	At 28 days	At 4 months
**25(OH) D (ng/ml)**	17.97 ± 9.35	17.82 ± 6.34	33.95 ± 13.4ª^,b^
**PTH (pg/mL)**	49.1(84.68)	39.6(92.63)	26.5(24.23) ^a,b^
**Osteocalcin (ng/mL)**	61(104.6)	98.05(58.8) ^a^	110.34(64) ^a^
**MMP-8 (ng/mL))**	500.8(70.3)	454.6(138.49)	1113(1338. 5) ^a,b^

PTH: Parathyroid hormone. MMP-8: Matrix metalloproteinase-8. Data are given as mean values ± standard deviation, and medians (interquartile range)

^a^ *p* < 0.05 value from baseline; ^b^ *p* < 0.05 value from 28 days

a) *Association between 25(OH) Vitamin D Levels at birth and comorbidities.*

The most frequent comorbidities were hyaline membrane disease (58%), LOS (48%) and BPD (24%). All the PTs with hyaline membrane disease were treated with surfactant following the neonatal unit protocol. The most prevalent proven sepsis blood culture was coagulase negative staphylococcus (21 cases) and 2 g negative (1 *Serratia marcescens* and 1 Klebsiella pneumonia). Considering a cut-off point of 20 ng/ml for 25(OH) D, 26 PTs (53%) had 25(OH) D serum levels < 20 ng/ml and 23 PTs (47%) had 25(OH) D levels > 20 ng/ml.

The incidence of comorbidities (bronchopulmonary dysplasia, hyaline membrane, necrotizing enterocolitis, and sepsis) did not differ in both groups (Table 5) when subdivided by vitamin D levels. However, when the two subgroups of PTs were arranged according to Vitamin D and PTH levels (25(OH) D < 20 ng/ml and PTH > 60 pg/ml versus 25(OH) D > 20 ng/ml and PTH < 60 pg/ml), the incidence of LOS was higher in the subgroup with vitamin D levels < 20 and PTH > 60 (RR 2.0, 95% CI: 1.3–3.55). We did not observe a higher incidence of other comorbidities. Comorbidity patterns in PTs with vitamin D levels measured at days 0 and 28 showed no differences except for the case of hyaline membrane, where vitamin D levels were unexpectedly higher both at birth and at 28 days (Table 6).

**Table 5:** Incidence of comorbidities during the first 28 days of life in preterm infants according to 25(OH) D levels at birth

	25(OH) D Baseline < 20 ng/ml	25(OH) D Baseline > 20 ng/ml	RR (IC 95%)
BPD	6(24%)	5(22.7%)	1.06 (0.66–1.71)
Hyaline Membrane Disease	13(50%)	16(69.6%)	0.72(0.4–1.29)
Retinopathy	2(8%)	1(4.3%)	1.84(0–4.7)
Necrotizing Enterocolitis	2(8%)	2(8%)	0.92(0.63–1.33)
Periventricular Leukomalacia	2(8%)	2(8%)	0.92 (0.63–1.33)
Intraventricular Hemorrhage	2(8%)	1(4.3%)	1.84 (0–4.7)
LOS	13(52%)	10(43.5%)	1.2 (0.36.3.95)

RR: Relative risk. Confidence Interval (CI) 95%. LOS: Late onset sepsis. BPD: bronchopulmonary dysplasia

**Table 6:** Relationship between 25(OH) D serum levels and comorbidities in Preterm Infants

	(N)	25(OH)D Baseline	p	25(OH)D 28 days	p
**BPD**	N [27]	17.8 ± 9.1	0.584	17.49 ± 6.65	0.532
	Y [12]	16.12 ± 7.71		18.88 ± 5.36	
**Hyaline Membrane** **Disease**	N [20]	14.32 ± 7.55	**0.04**	15.63 ± 5.33	**0.048**
	Y [28]	19.56 ± 8.99		19.36 ± 6.63	
**Retinopathy**	N [29]	17.4 ± 8.71	0.996	18.14 ± 6.23	0.192
	Y [3]	17.37 ± 11.06		13.17 ± 7.32	
**Necrotizing Enterocolitis**	N [30]	17.36 ± 8.62	0.917	17.61 ± 6.18	0.473
	Y [4]	17.84 ± 8.62		20.02 ± 8.58	
**Periventricular** **Leukomalacia**	N [30]	17.42 ± 8.86	0.96	17.94 ± 6.57	0.670
	Y [4]	17.19 ± 8.46		16.51 ± 3.18	
**Intraventricular Hemorrhage**	N [29]	17.57 ± 8.89	0.630	18.03 ± 6.42	0.4
	Y [3]	15.01 ± 6.97		14.8 ± 4.73	
**LOS**	N [26]	17.82 ± 8.99	0.738	19.74 ± 7.37	**0.031**
	Y [23]	16.94 ± 8.63		15.73 ± 4.23	

BPD: Bronchopulmonary dysplasia. LOS: Late onset sepsis. No: N; Yes: Y

Analysis of variance (ANOVA) with repeated measures for comparisons between groups. Data are expressed as mean ± SD

b) *Association between 25(OH) Vitamin D Levels al 28 days and LOS.*

It is worth mentioning that neonates with LOS had lower vitamin D levels on day 28 compared to those non-septic PTs (Table 6). Characteristics of PTs who developed LOS and those without this complication are described in table 7. At 28 days of life, we conducted a cross-sectional study, where 91% of PTs who developed LOS had 25(OH) D levels < 20 ng/ml. The logistic regression analysis was carried out with correction for newborn weight, mechanical ventilation, days of parenteral nutrition, CRIB I and CRIB II index as potential confounders. It was demonstrated that 25(OH) D levels at 28 days were independently associated with LOS (OR: 18.9;95% CI: 1.44–247.2; P: 0.025). MMP-8 levels showed no significant differences in PTs regarding LOS neither at birth (mean (IQR): 494.1 (3011.4) versus 504.7 (339.7); p: 0.188) nor at 28 days (470.8 (100.6) versus 454.2 (145.9) p: 0.817). We, furthermore, did not observe any correlation with C-reactive protein at any time-point.

**Table 7:** Baseline characteristics of Preterm Infants with and without LOS

	Preterm Infants LOS	
	Yes N:23	No N:26
**Gender (male)**	13(56%)	10(40%)
**Gestational age (weeks)**	29.57 ± 2.51	30.48 ± 2.08
**Newborn weight (gr)**	1197 ± 267.6	1435.16 ± 294.32^a^
**Crib I**	2(4)	0(1) ^a^
**Crib II**	7.76 ± 3.57	4.68 ± 2.95^a^
**Breastmilk feeding**	9(18%)	10 (20%)
**Parenteral nutrition/central catheter exposure (days)**	17 (8)	10 (4) ^a^
**Mechanical ventilation**	15(68%)	10(40%)
**Days of hospitalization**	48(35)	36(46) ^a^

LOS: Late onset sepsis; Cribs Clinical Risk Index for Babies score

Data are given as mean values ± standard deviation, and medians (interquartile range)

^a^ p < 0.05

## Discussion

The study of hypovitaminosis D has aroused great interest in recent years, as it relates to numerous pathologies in both adults and children, and, especially, in neonates. Our study was conducted in a single tertiary center and enrolled 50 Caucasian neonates from the same geographical area, with similar socioeconomic status, feeding and environmental conditions. In contrast to other studies, we observed that low vitamin D levels alone are not associated with prematurity or related comorbidities [28, 31–33]. However, a field of study opens when low 25(OH) D and high PTH levels are associated with a greater incidence of LOS in PTs.

Levels of 25(OH) D in cord blood were lower in term infants than in PTs, however, there were no significant differences. These results might have been influenced by our sample size. The percentage of vitamin D deficiency among the global population remains similar to what has been described by other authors [34, 35]. In our study, we have detected a higher percentage of vitamin D deficiency among term infants than in PTs, but such does not have statistical significance.

McDonnell SL et al. have reported that higher target levels of vitamin D must be achieved in pregnant women and their neonates to prevent adverse outcomes [36]. Despite having implemented vitamin D supplementation in PTs admitted to our neonatal unit, as shown elsewhere in this study, there were no significant differences regarding 25(OH) D serum levels in PTs at birth and at 28 days. Nevertheless, these levels increased significantly whereas PTH decreased also significantly at 4 months.

Vitamin D is responsible for increasing intestinal calcium absorption that feeds back the parathyroid gland to decrease PTH secretion. Exposure to comorbidities including hyaline membrane disease, NEC, patent ductus arteriosus and sepsis, among other pathologies, could explain the non-increase in vitamin D in PTs within the first 28 days of life. Consequently, PTs admitted to the NICU are at higher risk for vitamin D deficiency. On the other hand, defining hypovitaminosis D status in PTs and establishing a corresponding clinical significance is still a matter of controversy. To our knowledge, scarce is known about the PTH cut-off point related to 25 (OH) D in PTs [37, 38].

Maternal vitamin D stores have been the target of extensive scientific research, which has focused on their potential negative effects on maternal and neonatal health. Multiple observational studies have described that maternal hypovitaminosis D (maternal 25(OH) D serum levels < 20 ng/mL) is a crucial risk factor for neonatal adverse outcomes. Important differences in maternal and fetal outcomes have been reported when analysing pregnancy outcomes using serum biomarker 25(OH) D, with improved health in women who achieved circulating 25(OH) D serum levels of at least 40 ng/mL [39]. Moreover, recent major evidence reports that an effect of vitamin D deficiency is linked to a risk of miscarriage and some authors advocate for the introduction of level measurement before pregnancy [27].

Up to date, there is no consensus on the normal range for 25(OH) D serum levels in PTs and term infants. Hollis et al. [38] have suggested that pregnant women should have 25(OH) D serum levels > 40 ng/mL for optimizing maternal and neonatal outcomes. There is growing evidence that the higher vitamin D levels are, the fewer perinatal comorbidities, cesarean sections and hypertensive disorders will develop [15]. Mothers of both PTs and term infants showed no differences in blood pressure based on 25(OH) D serum levels.

Even though most prior studies highlight GA as a risk factor for vitamin D deficiency, we did not observe this in our study. Our findings, moreover, showed a correlation between maternal and neonatal 25(OH) D serum levels in PTs and term infants.

No mother received vitamin D supplementation during pregnancy, and vitamin D deficiency was prevalent in the recruited infants. Accordingly, previous studies have published a high prevalence of vitamin D deficiency in pregnant women [11, 40]. Unlike other studies, neither did we find higher 25(OH) D serum levels in term infants when compared to PTs nor even when participants in both groups had been born during the summer.

Vitamin D requirement during pregnancy is probably higher in the second and third trimester due to enhanced intestinal calcium absorption and fetal requirements [41]. Currently, there is no consensus on this issue, and different authors highlight the importance of vitamin D supplementation in pregnancy even though in our environment it is not a common practice. As a result, vitamin D supplementation during pregnancy should be considered when our aim is to minimize the danger of neonatal birth infections and improve maternal outcomes [42].

Several maternal risk factors which contribute to low maternal/fetal 25(OH) D serum levels have been reported, but no clear pattern has been established for multi-ethnic populations. The winter season, obesity, a lower socioeconomic status including lifestyle factors (smoking) and medication pose a risk for lower maternal/fetal transfer of vitamin D. Notwithstanding, there is still scarce published research into the relationship between some of the maternal risk factors and neonatal 25(OH) D serum levels [43]. In our study, factors such as the socioeconomic status, diet and lifestyle were similar for all the mothers. Parenteral nutrition could be considered a confounder factor related to the cumulative dosage of vitamin D. However, as parenteral nutrition is individualized since the first day of life, the amount of vitamin D administered to all the PTs is always the same (120 IU/day). The total amount of vitamin D was adjusted daily considering parenteral nutrition as well as feeding.

Critically ill patients have a high prevalence of vitamin D deficiency and low levels are associated with greater illness severity and morbidity [44]. It is likely that PTs have a compromised vitamin D status at birth and during NICU hospitalization. Xiaonan et al. [45] have reported that vitamin D deficiency is a risk factor for BPD in extremely PTs. Cetinkaya et al. [46] have found that the lower the maternal/neonatal 25(OH) D serum levels, the higher the risk for BPD development. We determined BPD based on the definition suggested by Jobe AH and Bancalari [25]. In contrast to other studies, we did not observe any relationship between 25(OH) D cord blood levels/28 days and risk for BPD. Joung KE et al. [30] have concluded that in extremely PTs neither cord blood nor the 36 weeks of corrected age for 25(OH) D serum levels are associated with BPD development. The lack of this relationship in our study may be accounted for the presence of a higher GA in our groups (median weeks’ GA 29.2 ± 2.45).

Recent studies in neonatal population have described improved outcomes including normalization of PTH at levels of vitamin D > 30 ng/mL. PTH is a major hormone in charge of bone resorption, and its serum levels may be a useful identification risk marker of secondary hyperparathyroidism and metabolic bone disease in extremely low birth weight neonates. In PTs, we had higher PTH levels at birth and these did not reach a normal range until 4 months. Our results indicate that the isolated determination of vitamin D does not define its deficiency or sufficiency and that the vitamin D-PTH relationship must be taken into consideration [29].

MMP-8 has been identified as a biomarker of neonatal sepsis [23]. The activity of MMP-8 would increase in many pathophysiological conditions such as severe infection [47]. Emerging evidence has supported the antimicrobial implications of vitamin D, as it enhances the innate immunity and induces the production of antimicrobial peptides that inhibit the growth of bacteria [48, 49]. Recently, Rosendahl et al. [50] have observed a positive correlation between 25(OH) D levels and MMP-8 in cord blood of healthy non-vitamin D deficient neonates. In our study, decreased serum levels of 25(OH) D neither correlated with MMP-8 levels nor with the incidence of early sepsis or LOS in PTs at any of the three-time points. However, we found a significant relationship between lower 25(OH) D cord blood levels and higher PTH with LOS in PTs (*p* < 0.031). In our PTs population, 25(OH) D levels in cord blood by itself did not represent an independent modifiable risk factor of lower morbidity related to sepsis.

The LOS incidence rate in PTs ranges between 20 and 38% in the first 120 days of life [51]. Prevalence of LOS is rather high in our study population (48%), which might be due to the fact that PTs were selected consecutively so as to avoid selection bias. On the other hand, the total number of days of central catheter exposure and of parenteral nutrition is a known risk factor for sepsis whereas human breastmilk administration is protective. As shown in table 7, there were neither differences in the rates of breastmilk feeding nor in exposure to mechanical ventilation. On this statement, differences detected among days on parenteral nutrition in the PTs who developed sepsis were related to the slower advances of enteral feeding in this group, resulting also in a longer hospital stay. Vitamin D levels are decreased after 28 days in patients who developed LOS.

In contrast to the findings reported by Fort et al. [52], after vitamin D supplementation with 1000 IU/day in our cohort of PTs during NICU hospitalization, biochemical 25(OH) D deficiency did not decrease within the first 28 days, and it was not until 4 months when values reached > 30 ng/mL, which associated with an optimal physiological function. Similar results have been reported by Cho et al. [53] in this regard. It is pivotal to establish the optimal amount of vitamin D intake among hospitalized PTs to achieve adequate vitamin D levels and prevent adverse events. However, the role of vitamin D in neonatal immunomodulation and the timing/dosage of vitamin D supplementation are still unknown and warrant further research.

The limitations of the present study include the difficulty to collect a homogeneous sample of Caucasian PTs and healthy term infants with the same environmental conditions. The selection bias was avoided by establishing very strict inclusion criteria for PTs ≤ 32 weeks’ GA and /or ≤ 1500 g birth weight in a single Neonatology Unit, using the same protocol for all the patients. The estimation of the sample size, recruited consecutively, provided sufficient power to detect differences and associations.

## Conclusions

In the present study, there was no significant relationship between the levels of vitamin D among the PTs ≤ 32 weeks’ GA and/or ≤ 1500 g birth weight and the term infants. Regarding comorbidities among the PTs, we solely detected a relationship between 25(OH) D serum levels < 20 ng/ml and PTH levels > 60 pg/mL and a higher incidence of LOS. At 28 days of life, more than 90% of the PTs who developed sepsis showed vitamin D levels < 20 ng/ml. Further research is warranted to establish the physiopathology and to determine whether a higher dose of vitamin D is required among PTs.
